# Modulation of Spike-Timing Dependent Plasticity: Towards the Inclusion of a Third Factor in Computational Models

**DOI:** 10.3389/fncom.2018.00049

**Published:** 2018-07-03

**Authors:** Alexandre Foncelle, Alexandre Mendes, Joanna Jędrzejewska-Szmek, Silvana Valtcheva, Hugues Berry, Kim T. Blackwell, Laurent Venance

**Affiliations:** ^1^INRIA, Villeurbanne, France; ^2^LIRIS UMR 5205 CNRS-INSA, University of Lyon, Villeurbanne, France; ^3^Dynamic and Pathophysiology of Neuronal Networks, Center for Interdisciplinary Research in Biology (CIRB), College de France, INSERM U1050, CNRS UMR7241, Labex Memolife, Paris, France; ^4^University Pierre et Marie Curie, ED 158, Paris, France; ^5^The Krasnow Institute for Advanced Studies, George Mason University, Fairfax, VA, United States

**Keywords:** STDP, third factor, dopamine, acetylcholine, noradrenaline, astrocytes, eligibility traces, Hebbian plasticity

## Abstract

In spike-timing dependent plasticity (STDP) change in synaptic strength depends on the timing of pre- vs. postsynaptic spiking activity. Since STDP is in compliance with Hebb’s postulate, it is considered one of the major mechanisms of memory storage and recall. STDP comprises a system of two coincidence detectors with N-methyl-D-aspartate receptor (NMDAR) activation often posited as one of the main components. Numerous studies have unveiled a third component of this coincidence detection system, namely neuromodulation and glia activity shaping STDP. Even though dopaminergic control of STDP has most often been reported, acetylcholine, noradrenaline, nitric oxide (NO), brain-derived neurotrophic factor (BDNF) or gamma-aminobutyric acid (GABA) also has been shown to effectively modulate STDP. Furthermore, it has been demonstrated that astrocytes, via the release or uptake of glutamate, gate STDP expression. At the most fundamental level, the timing properties of STDP are expected to depend on the spatiotemporal dynamics of the underlying signaling pathways. However in most cases, due to technical limitations experiments grant only indirect access to these pathways. Computational models carefully constrained by experiments, allow for a better qualitative understanding of the molecular basis of STDP and its regulation by neuromodulators. Recently, computational models of calcium dynamics and signaling pathway molecules have started to explore STDP emergence in *ex* and *in vivo*-like conditions. These models are expected to reproduce better at least part of the complex modulation of STDP as an emergent property of the underlying molecular pathways. Elucidation of the mechanisms underlying STDP modulation and its consequences on network dynamics is of critical importance and will allow better understanding of the major mechanisms of memory storage and recall both in health and disease.

## Introduction

Most computational and experimental studies of synaptic plasticity focus on variations of Hebb’s rule in which the change in synaptic strength is caused by direct association of two factors, i.e., two inputs (or activity patterns), one on the presynaptic and one on the postsynaptic side. Thus, when neural circuits adjust their synaptic weights depending on the frequency or timing of the pre-synaptic and post-synaptic firing patterns, Hebb’s postulate is fulfilled. In addition, a third factor (for example neuromodulators or astrocytes) stabilizes or modulates the expression of synaptic plasticity and, thus, ultimately learning (Kempter et al., 1998; Pawlak et al., 2010; Lisman et al., 2011; Frémaux and Gerstner, 2016; Edelmann et al., 2017; Kuśmierz et al., 2017; Gerstner et al., 2018). The inclusion of this third factor with two-factor Hebbian plasticity rule is called neoHebbian plasticity (Lisman et al., 2011), and is infrequent in computational models of spike-timing dependent plasticity (STDP). In this review article, we focus on STDP (Sjöström et al., 2008; Feldman, 2012), a synaptic Hebbian learning rule, and its control by the third factor: neuromodulation (via the action of dopamine, acetylcholine, noradrenaline and others) or astrocyte activity. Our goal is to highlight aspects of STDP that should be taken into account in future computational models of STDP.

Since its discovery, STDP has attracted considerable interest in experimental and computational neuroscience because it avoids implausibly high firing frequencies and instead relies on spike correlation. STDP has emerged as a candidate mechanism for experience- and activity-dependent changes in neural circuits, including map plasticity (Abbott and Nelson, 2000; Dan and Poo, 2006; Morrison et al., 2008; Sjöström et al., 2008; Feldman, 2012; Froemke, 2015). Experiments in different brain regions and in diverse neuronal types have revealed a plethora of STDP forms that vary in plasticity direction, temporal dependence and the involvement of signaling pathways (Sjöström et al., 2008; Feldman, 2012; Korte and Schmitz, 2016). Experimental protocols that investigate STDP use pairing of a presynaptic stimulation with a postsynaptic spike, with the pre- and postsynaptic stimulations separated by a fixed interval Δt_STDP_(spike timing). In most of the studies, the spike timing is computed as Δt_STDP_ = t_post_−t_pre_, where t_post_ and t_pre_ are the times of emission of the postsynaptic spike and that of the presynaptic stimulation, respectively. If the postsynaptic stimulation occurs before the presynaptic, Δt_STDP_ < 0 (post-pre pairings), whereas Δt_STDP_ > 0 when the presynaptic stimulation occurs before the postsynaptic one (pre-post pairings). The same pairing pattern is then repeated between 50 and 200 times at a constant frequency (typically between 0.1 Hz and 5 Hz). The canonical STDP is bidirectional (able to generate potentiation and depression depending on the value of Δt_STDP_) and Hebbian, i.e., post-pre pairings (Δt_STDP_ < 0) yield timing-dependent long-term depression (tLTD) and pre-post pairings (Δt_STDP_ > 0) give rise to timing-dependent long-term potentiation (tLTP). For most STDP forms, the expression of plasticity is restricted to a narrow temporal window (|Δt_STDP_| < 80 ms); thus, when pre- and postsynaptic activities are separated by a large Δt_STDP_, long-term synaptic changes are not observed (Markram et al., 1997; Bi and Poo, 1998).

The predominant form of STDP is Hebbian, and has been observed in the neocortex (Markram et al., 1997; Feldman, 2000; Sjöström et al., 2001; Froemke et al., 2005; Nevian and Sakmann, 2006), the hippocampus (Debanne et al., 1997, 1998; Bi and Poo, 1998; Nishiyama et al., 2000; Wittenberg and Wang, 2006), and the striatum (Fino et al., 2008, 2009; Pawlak and Kerr, 2008; Shen et al., 2008). In contrast to Hebbian STDP, bidirectional anti-Hebbian STDP expresses tLTP for Δt_STDP_ < 0 and tLTD for Δt_STDP_ > 0. Anti-Hebbian STDP was first reported in the cerebellum-like structure of electrical fish (Bell et al., 1997). More recently, bidirectional anti-Hebbian STDP has been observed in mammals and in various structures including the striatum (Fino et al., 2005, 2010; Schulz et al., 2010; Paille et al., 2013; Valtcheva et al., 2017) and the somatosensory cortex (Letzkus et al., 2006). Unidirectional anti-Hebbian forms of STDP inducing tLTD for both Δt_STDP_ < 0 and Δt_STDP_ > 0, have been observed in the cerebellum (Han et al., 2000; Safo and Regehr, 2008), the neocortex (Egger et al., 1999; Lu et al., 2007), the dorsal cochlear nucleus (Tzounopoulos et al., 2004) and the hippocampus (Wittenberg and Wang, 2006). Recently, a unidirectional Hebbian STDP where tLTP was observed for both post-pre and pre-post pairings, has been reported in hippocampus (Mishra et al., 2016). The mechanisms that produce these diverse forms of STDP are not completely understood, though could involve a third factor, such as neuromodulators (such as dopamine or acetylcholine; for reviews see Pawlak et al., 2010; Edelmann et al., 2017) or astrocytes.

All the forms of STDP described so far depend on one of three main systems of coincidence detectors (Feldman, 2012; Korte and Schmitz, 2016). The first system comprises the N-methyl-D-aspartate receptor (NMDAR) receptor (NMDAR) as the unique coincidence detector for both tLTP and tLTD, though voltage-sensitive calcium channels may play a role in coincidence detection. This form of plasticity has been reported in hippocampal CA1 neurons (Nishiyama et al., 2000), neocortical layer 2/3 pyramidal cells (Froemke et al., 2005), striatal output neurons (Pawlak and Kerr, 2008) and striatal gamma-aminobutyric acid (GABA)ergic interneurons (Fino et al., 2008). The second system combines NMDAR-dependent tLTP with tLTD which depends on metabotropic glutamate receptor (mGluR)- and/or cannabinoid type-1 receptor (CB_1_R)-activation. Though the tLTD is independent of postsynaptic NMDARs, the activation of presynaptic NMDARs can be implicated (Sjöström et al., 2003; Bender et al., 2006; Corlew et al., 2007; Rodríguez-Moreno and Paulsen, 2008). This form of plasticity has been observed in the visual (layer 2/3) and somatosensory (layer 5) cortex (Sjöström et al., 2003; Bender et al., 2006; Nevian and Sakmann, 2006; Corlew et al., 2007; Rodríguez-Moreno and Paulsen, 2008), cholinergic striatal interneurons (Fino et al., 2008) or striatal output neurons (Fino et al., 2010). Recently in striatal output neurons, a third system has been reported, in which the tLTD is CB_1_R-dependent, whereas the molecular dependence of tLTP is governed by the number of pairings: a small number of pairings (~10) produces a CB_1_R-mediated tLTP, whereas greater number of pairings yields an NMDAR-mediated tLTP (Cui et al., 2015, 2016).

The molecular mechanisms accounting for these various forms of STDP are not yet fully understood, despite a substantial number of studies focusing on STDP. For the NMDAR-dependent tLTP and tLTD, calcium amplitude seems to partly determine plasticity direction (Nevian and Sakmann, 2006). For Δt_STDP_ > 0, when the presynaptic activity precedes the back-propagating action potential, the excitatory post-synaptic potential coincides with the back-propagating action potential resulting in high and more prolonged calcium influx through the NMDAR and voltage-sensitive calcium channels, which leads to tLTP. For Δt_STDP_ < 0, calcium influx through the NMDARs and voltage-sensitive calcium channels is lower and as a result induces tLTD (Magee and Johnston, 1997; Koester and Sakmann, 1998; Nevian and Sakmann, 2006; Pawlak and Kerr, 2008). These different calcium dynamics produce different directions of plasticity by recruiting different downstream signaling molecules. Several computational models have used a description of neuronal calcium dynamics and/or the kinetics of downstream signaling pathways as a proxy to predict the direction of plasticity (tLTP or tLTD). These computational models investigate the impact of different STDP timings or of modulators on STDP by integrating their effects on calcium dynamics or downstream signaling pathways. Therefore computational models based on the kinetics of the implicated molecular pathways are promising avenues to integrate the third factor in Hebbian plasticity and will be the main focus of the present review.

## Neuromodulators Affecting the Expression, Polarity and Shape of STDP

Neuromodulators and neurotransmitters play an important, but often unappreciated, role in the control of STDP induction and maintenance (for reviews see Pawlak et al., 2010; Edelmann et al., 2017). The skepticism about neuromodulation stems from the apparent discrepancy between the time scale of neuromodulation and the coincidence detection timing inherent to STDP. The former is on the scale of seconds or more, whereas the latter is on the scale of milliseconds. However, this apparent discrepancy becomes less important after considering STDP from the perspective of a learning system that needs to link recorded information (memory) with a value scale (reward). Indeed, an individual acting on its environment needs to learn to discriminate actions leading to reward from those leading to punishment, both possibly occurring seconds, minutes or even hours after the taken action. A system of memory and learning based only on the timescale of STDP would miss this essential information. Thus, one role of neuromodulation is to link STDP and the reward system. In this context, we demonstrate below how a third factor, comprised of neuromodulators and/or astrocytes, modulates the timing dependence of STDP. Note that the modulation of timing dependence depends on brain region and cell type; thus future computational models will need to incorporate region and cell type specific modulation. In this section, we detail STDP protocols used in experimental studies because depending on the activity patterns neuromodulatory systems are differentially recruited. Therefore, the apparent contradiction between several of the experimental reports on STDP could depend on the activity patterns or neuromodulatory activation that were used. This knowledge might help the building of computational models, by taking into account the different regimes of action of neuromodulators in shaping STDP.

### Dopamine

**Figure 1:**
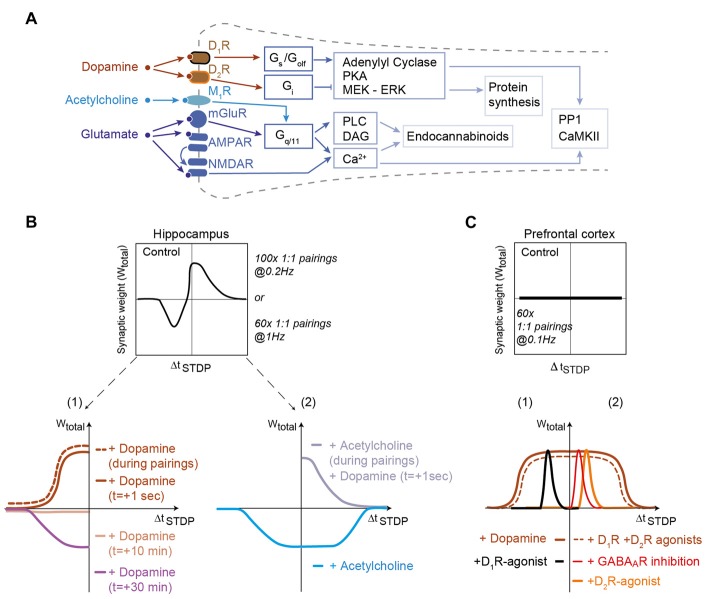
Dopamine and acetylcholine shape spike-timing dependent plasticity (STDP) in hippocampus and prefrontal cortex. **(A)** Generic schematics of the main signaling pathways activated in STDP in response to dopamine, acetylcholine and glutamate. Full and tee-shaped arrows denote activation and inhibition, respectively. G_x_, G-protein coupled receptor signaling x subclass; PKA, Protein kinase A, MEK-ERK (activation of MAPK); PP1, Protein Phosphatase-1; CaMKII, Ca^2+^/calmodulin-dependent protein kinase-II; DAG, diacylglycerol; PLC, phospholipase C. **(B)** In hippocampus, bidirectional Hebbian STDP observed in control conditions is converted to timing-dependent long-term potentiation (tLTP) when dopamine is applied during the STDP pairings or just after it. When dopamine is applied 10 and 30 min after STDP pairings, an absence of plasticity and timing-dependent long-term depression (tLTD) are observed, respectively. Adapted from Zhang et al. (2009) and Brzosko et al. (2015). Acetylcholine, applied during STDP pairings, converts bidirectional Hebbian STDP to unidirectional tLTD for both post-pre and pre-post pairings. Dopamine applied just after STDP pairings with acetylcholine during STDP pairings can rescue pre-post tLTP. Adapted from Brzosko et al. (2017). **(C)** In the prefrontal cortex, addition of dopamine or D_1_-plus D_2_-class receptor agonists to a pairing protocol that does not induce STDP promotes a unidirectional tLTP. The inhibition of GABA_A_ receptors or application of agonists of D_2_-class receptors allows the expression of tLTP for pre-post pairings. Conversely, application of agonists of D_1_-class receptors allows the expression of tLTP for post-pre pairings. Activation of D_2_R expressed by gamma-aminobutyric acid (GABA)ergic interneurons (or their direct inhibition by GABA_A_ receptor inhibitors) decreases activity of these interneurons uncovering tLTP for pre-post pairings. For post-pre pairings induction relies on D_1_-class receptor (located on the postsynaptic neuron) activation. Adapted from Xu and Yao (2010) and Ruan et al. (2014), with no permission required.

The action of dopamine is mediated by the metabotropic dopaminergic receptors that functionally modulate other receptor systems and/or ion channels without inducing large postsynaptic currents. Dopaminergic receptors belong to two groups based on their G-protein coupling: the D_1_-class receptors (D_1_R and D_5_R) are coupled to Gs- or G_olf_-proteins and the D_2_-class receptors (D_2_R, D_3_R and D_4_R) to Gi/o-proteins (Neve et al., 2004). D_1_- and D_2_-class receptors have opposite action on the cyclic adenosine monophosphate (cAMP) second messenger pathway and the protein kinase A (PKA; Figure 1A).

Dopamine is released by midbrain dopaminergic neurons in response to both reward and the reward prediction error (Schultz, 2007). In the hippocampus, tLTD, which is observed in control conditions for negative Δt_STDP_, is converted to tLTP by dopamine addition during STDP pairings or immediately after STDP pairings (aiming at mimicking a retroactive effect; Zhang et al., 2009; Brzosko et al., 2015; Figure 1B). Dopamine addition during STDP induction leads to the enlargement of the temporal window of tLTP expression (Figure 1B). However the effects of dopamine disappear when dopamine is added long after STDP pairings, since dopamine addition 10 and 30 min after pairings results in an absence of plasticity and a recovery of tLTD observed in control conditions, respectively (Brzosko et al., 2015; Figure 1B). This dopaminergic modulation, which converts bidirectional STDP to unidirectional tLTP, is D_1_R- but not D_2_R-mediated (Zhang et al., 2009; Brzosko et al., 2015). Acetylcholine (classically associated with arousal and exploratory behavior; Ma et al., 2018) transforms bidirectional Hebbian hippocampal STDP into unidirectional tLTD (Brzosko et al., 2017). However, the effect of acetylcholine is reverted by dopamine addition 1 s after STDP pairings, which allows recovering tLTP (Figure 1B). Although these results constitute an important step for the experimental demonstration of a retroactive action of dopamine on Hebbian plasticity, the molecular mechanisms underlying dopamine interactions with the coincidence detectors were not characterized. In addition, more distal action of dopamine from STDP protocol remains to be investigated to fully explore the temporal credit-assignment problem (Sutton and Barto, 1998; Izhikevich, 2007; Schultz, 2007; Gerstner et al., 2018).

Additional evidence supports the role of dopamine for promoting hippocampal tLTP. Conditions that lower basal dopamine during the preparation of brain slices prevent the induction of tLTP at synapses between Shaffer collaterals and CA1 pyramidal cells (Edelmann and Lessmann, 2011). Subsequent addition of dopamine rescues tLTP, through a D_1_R-mediated mechanism (Edelmann and Lessmann, 2011, 2013). In addition, D_1_- and D_5_R-activations are important for the induction of tLTP at the synapses between the medial perforant pathway and dentate gyrus neurons (Yang and Dani, 2014). The mechanism here includes a change in cell excitability: inactivation of the transient A-type potassium current by D_1_R and D_5_R increases the excitability of dentate gyrus neurons and the amplitude of their back-propagating action potentials (Yang and Dani, 2014).

Beyond the hippocampus, the importance of dopamine modulation of STDP also is attested in the basal ganglia, where dopamine plays a crucial role in motor control, action selection and reinforcement learning (Yin and Knowlton, 2006; Schultz, 2007). Given the importance of dopamine, it is not surprising that dopamine is required for STDP in the striatum, both *ex vivo* (Pawlak and Kerr, 2008; Shen et al., 2008) and *in vivo* (Schulz et al., 2010; Fisher et al., 2017). However, the situation is complicated by the diversity in dopamine receptors. In rodents, striatal output neurons belong either to the direct or the indirect trans-striatal pathways and show different dopaminergic receptor expression, D_1_- and D_2_-class receptors, respectively (Calabresi et al., 2014). *In vivo* in anesthetized rodents, negative and positive pairing STDP protocol both result in tLTD at corticostriatal synapses, and bidirectional STDP can be elicited only with phasic dopaminergic release obtained by electrical stimulation of midbrain dopaminergic neurons (Schulz et al., 2010) or pharmacological manipulation of dopaminergic transmission (together with GABAergic and adenosine transmissions; Fisher et al., 2017). These results are consistent with *ex vivo* studies, which showed that application of dopamine either simultaneously, or 0.6 s after glutamate allows dendritic spine enlargement and calcium increase (Yagishita et al., 2014). Moreover, this study demonstrated the existence of synaptic eligibility traces, which can be revealed by subsequent dopamine release after Hebbian learning (see section “Monoamines Transform Eligibility Traces Into Plasticity” below).* Ex vivo*, conflicting results have been reported regarding STDP modulation by dopamine: according to Pawlak and Kerr (2008) both tLTD and tLTP requires D_1_R- but not D_2_R-activation (D_2_R-activation affecting only plasticity kinetics: tLTP and tLTD onset is shortened and delayed, respectively), whereas Shen et al. (2008) reported that D_2_R-activation is required for tLTD expression in striatal neurons belonging to the indirect pathway and D_1_R-activation is necessary for tLTP in striatal neurons belonging to the direct pathway. There are methodological differences between these two studies which could account for this discrepancy in results: for post-pre and pre-post pairings the same STDP protocol (i.e., 100 pairings at 0.1 Hz) was applied by Pawlak and Kerr (2008), whereas two distinct STDP-like protocols (theta bursts 3:3 for tLTP and 1:3 for tLTD) were utilized by Shen et al. (2008). Depending on the activity patterns, D_1_- and D_2_-class receptors could be differentially activated. The effects of dopamine in the striatum via D_2_R receptors would result from a D_2_R-mediated attenuation of both synaptic- and back-propagating action potential-evoked calcium influx into dendritic spines via the inhibition of PKA-dependent regulation of NMDARs (Higley and Sabatini, 2010; Figure 1). This mechanism also is supported by the demonstration that dopamine depletion enhances calcium influx in dendrites of the D_2_R-expressing striatal neurons belonging to the indirect pathway (Day et al., 2008). Future development of detailed computational models of the signaling pathways will be useful for fully exploring the involvement of dopaminergic receptors in various forms of STDP (see “Molecular Pathway-Based Computational Models of STDP” section).

The role of dopamine has been demonstrated in two other brain regions, the prefrontal cortex and the amygdala. In the prefrontal cortex (at layer 5 pyramidal cells) an STDP protocol such as 60 pairings (Δt_STDP_ = +10 ms) at 0.1 Hz fails to produce plasticity, while dopamine application during the STDP pairings permits the induction of Hebbian tLTP (Δt_STDP_ = +10 ms; Xu and Yao, 2010) and anti-Hebbian tLTP (Δt_STDP_ = −30 ms; Ruan et al., 2014; Figure 1B). Both Hebbian and anti-Hebbian tLTP directly depends upon D_1_R-activation in the postsynaptic neuron whereas the Hebbian tLTP depends also indirectly upon the activation of D_2_R expressed by GABAergic interneurons. D_2_R activation blocks the inhibition exerted by GABAergic interneurons and permits the expression of Hebbian tLTP (Δt_STDP_<+10 ms). By combining D_1_R- and D_2_R-activation, the temporal window of tLTP is extended up to Δt_STDP_ = +30 ms (Xu and Yao, 2010; Figure 1C). This suggests that in prefrontal cortex, the physiological form of STDP is the anti-Hebbian tLTP since the expression of Hebbian tLTP is disfavored by GABAergic network activity. In the lateral nucleus of the amygdala tLTP requires the activation of D_2_R located on neighboring GABAergic interneurons (Bissière et al., 2003). Since dopamine is released in the amygdala in response to stress (Inglis and Moghaddam, 1999), dopaminergic neuromodulation of inhibitory synaptic transmission appears to be a crucial mechanism underlying the acquisition of fear conditioning.

In summary, these results show that dopamine is a key neuromodulator of STDP and constitutes the third factor required for the temporal credit-assignment. Overall, the effects of dopamine seem to conform to a simple general scheme: the activation of G_s_/G_olf_-coupled D_1_R tends to promote tLTP whereas the activation of G_i_-coupled D_2_R favors tLTD. However, the effects exerted by dopamine strongly depend on the brain area: dopamine either can be mandatory for STDP induction and/or maintenance or modulate STDP properties (width of the Δt_STDP_ window, polarity of the STDP or magnitude of the plasticity). Moreover, network effects can add complexity to the picture, since the expression of dopamine receptors is not restricted to the examined neuron but can affect the response to e.g., local interneurons.

### Noradrenaline

**Figure 2:**
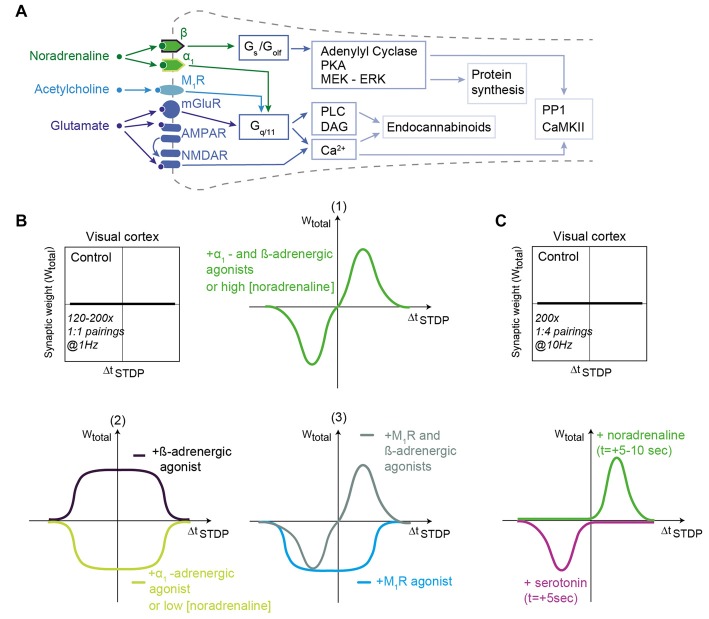
Noradrenaline and acetylcholine shape STDP in the visual cortex. **(A)** Generic schematic of the main signaling pathways activated in STDP in response to noradrenaline, acetylcholine and glutamate. Abbreviations are those of Figure 1A. **(B)** In layer 2/3 of the visual cortex, STDP protocols consisting of 120–200 pairings at 1 Hz do not produce STDP in control conditions. When α1- and β-adrenergic receptor agonists (1) or when muscarinic M_1_R and β-adrenergic receptor agonists (3) are applied, then bidirectional Hebbian STDP can be observed. Unidirectional anti-Hebbian tLTD and unidirectional Hebbian tLTP are induced after α1- and β-adrenergic receptor agonist application, respectively (2); M_1_R agonist promotes unidirectional anti-Hebbian tLTD (3). Low and high concentration of noradrenaline promote unidirectional anti-Hebbian tLTD (2) and bidirectional Hebbian STDP (1), respectively. **(C)** Monoamines transform eligibility traces into plasticity. Hebbian pairings (200 pairings at 10 Hz) induce post-pre tLTD and pre-post tLTP only if serotonin and noradrenaline are released 5–10 s after STDP pairings. Adapted from Seol et al. (2007), Salgado et al. (2012), Guo et al. (2012), Huang et al. (2013) and Huang et al. (2014), He et al. (2015) with no permission required.

Noradrenaline interacts with G-protein–coupled receptors of three families: α2-, α1- and β1-3-adrenergic receptors (by order of decreasing affinity; Ramos and Arnsten, 2007). α2-adrenergic receptors are Gi/Go-coupled and lead to cAMP decrease. α1-adrenergic receptors are Gq-coupled, and activate phospholipase Cβ (PLCβ), resulting in intracellular calcium release via inositol 1,4,5-triphosphate (IP3). β-adrenergic receptors are Gs-coupled and yield cAMP increase (Figure 2A).

In several brain regions, activation of adrenergic receptors modifies the shape of the STDP curve (Figure 2B). In the hippocampus, activation of β-adrenergic receptors enlarges the range of Δt_STDP_ for Hebbian tLTP expression by increasing the excitability of CA1 pyramidal cells (Lin et al., 2003). In the visual cortex, whereas paired stimulations of layer 4 afferents with postsynaptic action potential bursts does not produce plasticity, the concomitant activation of adrenergic receptors (with both α1-and β-adrenergic receptor agonists) allows the emergence of bidirectional Hebbian STDP in pyramidal cells of layer 2/3 (in rodents: Guo et al., 2012; in primates: Huang et al., 2014; Figure 2B) as well as in fast-spiking interneurons and non-fast-spiking somatostatin-positive interneurons (Huang et al., 2013). Note that α1-and β-adrenergic receptor agonists alone (or agonists of M_1_-class muscarinic acetylcholine receptors (mAChRs), see section below; Seol et al., 2007) trigger a tLTD-only (i.e., unidirectional anti-Hebbian STDP), whereas β-adrenergic receptor agonists alone induce the expression of a tLTP-only (i.e., unidirectional Hebbian STDP; Seol et al., 2007; Guo et al., 2012; Huang et al., 2013; Figure 2B). The affinity for noradrenaline of α1-adrenergic receptors exceeds that of β-adrenergic receptors, and unidirectional anti-hebbian STDP (tLTD-only) is observed in low noradrenaline, whereas bidirectional Hebbian STDP can be induced with higher noradrenaline concentration (Salgado et al., 2012; Figures 2B,C).

Taken together, those studies show that adrenergic receptors play an important role in shaping STDP, mostly by enlarging Δt_STDP_ and controlling STDP polarity, but also, similarly to dopamine, by acting subsequently to the stimulation to promote plasticity. Overall, a pattern emerges from the effects of noradrenaline: the activation of Gs-coupled β-adrenergic receptors tends to promote tLTP, whereas the activation of Gq-coupled α1-adrenergic receptors tends to favor tLTD.

### Monoamines Transform Eligibility Traces Into Plasticity

One of the fundamental questions in reward learning is the temporal credit-assignment problem: how are the correct actions learned given that delivery of a reward or punishment occurs significantly later than the key actions that promoted the outcome (Schultz, 2007). In an attempt to solve the temporal credit-assignment problem, some computational studies addressed the question of the retroactive effect of dopamine on cortical and hippocampal STDP (Sutton and Barto, 1998; Izhikevich, 2007; Gerstner et al., 2018). From a cellular perspective, the temporal credit-assignment problem translates into the following question: if dopamine (and more broadly monoamines) modulates STDP, is there a dependence of this modulation on the time elapsed between the stimulus (STDP pairings) and the reward (release of monoamines)? This question adds a supplementary temporal dimension to the modulation by the third-factor monoamine.

To solve the temporal credit-assignment or the distal reward problem, it has been proposed that synaptic eligibility traces could constitutes synaptic tags that are set by Hebbian learning and that will be transformed subsequently into synaptic plasticity by neuromodulators, bridging the learning sequence with reward (Sutton and Barto, 1998; Izhikevich, 2007; Gerstner et al., 2018). In other words, eligibility traces would be induced by Hebbian learning but would remain silent in term of synaptic efficacy changes, unless a neuromodulator released subsequently transforms them for plasticity. Synaptic eligibility traces would allow the synapse to keep a trace from the stimulus until getting the reward, the latter of which is represented by monoamines. We can distinguish two cases: the subsequent release of neuromodulator shapes an existing plasticity (Cassenaer and Laurent, 2012; Brzosko et al., 2015, 2017; Shindou et al., 2018) or allows the plasticity expression (Yagishita et al., 2014; He et al., 2015).

Octopamine, the equivalent of noradrenaline in insects, changes the bidirectional Hebbian STDP at synapses of Kenyon cells in the locust, critical for the associative learning of odors, into a unidirectional STDP (tLTD-only) even in a retroactive manner when applied seconds after the relevant pairing (Cassenaer and Laurent, 2012). In a similar way, in rodents, when dopamine is applied just after STDP pairings, it converts tLTD into tLTP in hippocampus (Brzosko et al., 2015, 2017) or in striatum (Shindou et al., 2018).

In striatum, dopamine induces spine enlargement exclusively when opto-stimulation of dopaminergic terminals occur between 0.3–2 s after Hebbian learning (i.e., STDP pairings; Yagishita et al., 2014). In the visual cortex and in the medial prefrontal cortex, release of noradrenaline and serotonin, just after the whole set of pairings or just after every pairing, allows the expression of tLTP and tLTD for pre-post and post-pre pairings, respectively (He et al., 2015); the STDP pairings *per se* did not induce plasticity (Figure 2C). He et al. (2015) observed that the eligibility traces are short-lived since the monoamines need to be release 5–10 s after learning to promote plasticity (He et al., 2015). The fact that a couple of monoamines (or third factors) is at play for distinct induction plasticity (tLTP vs. tLTD) could allow an efficient stabilization of learning and avoid synaptic saturation.

### Acetylcholine

Acetylcholine acts on two types of muscarinic receptors mAChRs: the M_1_-(M_1_, M_3_ and M_5_) and M_2_-(M_2_ and M_4_) class receptors (Thiele, 2013), and the ionotropic (cationic) nicotinic acetylcholine receptors (nAChRs; Albuquerque et al., 2009). M_1_-class mAChRs are Gq/G11-coupled leading to IP3 and diacylglycerol (DAG) production (via PLCβ activation), subsequent increase of intracellular calcium and activation of protein kinase C; (PKC; Figure 2A); M2-class mAChRs are Gi/Go-coupled, leading to inhibition of adenylate cyclase, and a reduction of cAMP and thus PKA activity.

Unlike STDP experiments with noradrenaline and dopamine, experiments to characterize the effect of acetylcholine have not carefully delineated M_1_-class vs. M_2_-class effects; thus experimental results are more diverse. At hippocampal CA1 pyramidal cells, bidirectional Hebbian STDP is converted into unidirectional tLTP after enhancement of acetylcholine (Brzosko et al., 2017; Figure 1B), whereas inhibition of mAChRs prevents post-pre tLTD and converts pre-post tLTP into tLTD (Sugisaki et al., 2011, 2016). When excitatory and inhibitory post-synaptic currents were examined at synapses of CA1 pyramidal neurons, pre-post pairings induce tLTP of excitatory pathway while it triggers tLTD at inhibitory pathways via the co-activation of mAChRs and CB_1_R (Ahumada et al., 2013). Thus, Hebbian STDP in CA1 pyramidal neurons depends on the excitation/inhibition balance, which is tightly regulated by mAChRs expressed in GABAergic interneurons and pyramidal cells (Sugisaki et al., 2011, 2016; Ahumada et al., 2013; Takkala and Woodin, 2013).

Though acetylcholine alone seems to promote unidirectional plasticity (tLTP- or tLTD-only), co-activation of mAChRs and Gs coupled pathways (either D1/D5 dopaminergic receptors in the hippocampus CA1 pyramidal cells (Brzosko et al., 2017) or β-adrenergic receptors in visual cortex layer 2/3 pyramidal cells (Seol et al., 2007) promotes bidirectional plasticity by restoring Hebbian tLTP for Δt_STDP_ > 0 (Figure 2B).

The effects of acetylcholine via nAChR-activation are expected to include depolarization and possibly increased calcium influx (Jones et al., 2012), but they also can exert a more subtle influence on STDP by regulating the magnitude of STDP rather than its polarity or expression (Sugisaki et al., 2016). Nicotine increases the threshold for the induction of Hebbian tLTP at excitatory synapses of pyramidal cells of the prefrontal cortex (Couey et al., 2007). However, note that nicotine when applied at a high concentration (~10 μM) can exert a more drastic effect on STDP since it converts tLTP into tLTD (Couey et al., 2007). Interestingly, in the medial prefrontal cortex, after nicotine treatment in juvenile rats, opposing effects are obtained depending on the developmental stage: tLTP magnitude was reduced in juvenile whereas it was increased in adult rats (Goriounova and Mansvelder, 2012).

Taken together, the above results reveal a general principle whereby the neuromodulatory effects exerted on STDP by monoamines (dopamine or noradrenaline) or acetylcholine are for a large part guided by the type of G-protein activated (regardless of the agonist): Gi/o-coupled and Gq/11-coupled receptor activation facilitates tLTD (D_2_-class, α1-adrenergic, M_1_-class), whereas Gs- and Golf-coupled receptor activation rather leads to the expression of tLTP (D_1_R-class, β-adrenergic receptors). However the validity of this general principle needs further investigation in other brain areas and neuronal subtypes.

### Brain-Derived Neurotrophic Factor (BDNF)

The neurotrophic factor brain-derived neurotrophic factor (BDNF) binds to the tyrosine receptor kinase B, which induces tyrosine receptor kinase B dimerization and the autophosphorylation of tyrosine residues in the cytoplasmic kinase domain. This process induces the activation of three main signaling pathways: phospholipase Cγ, phosphoinositide 3-kinase and extracellular signal-regulated protein kinases cascades. Notably, the phosphoinositide 3-kinase signaling pathway plays an important role in the regulation of mRNA translation, which impacts protein synthesis and putatively BDNF-dependent plasticity. Numerous studies have shown the role of BDNF in modulating synaptic transmission and plasticity (for reviews see Park and Poo, 2013; Edelmann et al., 2014).

Concerning STDP, pairings of glutamate release and postsynaptic spiking at 1 Hz are sufficient to release BDNF from the postsynaptic dendrites in a spike-timing-dependent manner (for 0 < Δt_STDP ≤ +20 ms; for Δt_STDP_ > 20 ms BDNF release was not detected; Lu et al., 2014). This spike-timing-dependent BDNF release is dependent on the activation of NMDARs. In hippocampal neurons, the tLTP part of the observed bidirectional Hebbian STDP depends on BDNF (Bi and Poo, 1998; Lu et al., 2014). Interestingly, depending of the activity pattern during STDP pairings, the BDNF dependence of the observed plasticity is different. Indeed, hippocampal tLTP induced with presynaptic activation paired with postsynaptic bursts of four back-propagating action potentials (1:4 pairings repeated 30 times at 0.5 Hz) is BDNF and tyrosine receptor kinase B-mediated, whereas canonical STDP pairings (1:1 pairings repeated 100 times at 0.5 Hz) induced a tyrosine receptor kinase B-independent tLTP at the same synapses (Edelmann et al., 2015). Genetic impairment of BDNF synthesis has led to alteration of STDP in the prefrontal cortex. Disruption of one of the promoters involved in BDNF transcription (promoter IV mutant mice) leads to the aberrant induction of tLTP, which is absent in wild-type mice, for 50 pairings (Sakata et al., 2009). In the infralimbic medial prefrontal cortex, STDP is absent in a rodent model (BDNF-Met/Met mice) of the human BDNF Val66Met polymorphism (leading to severe cognitive dysfunction and anxiety disorders) in which the BDNF release is impacted; STDP is recovered after exogenous BDNF application (Pattwell et al., 2012).

### Nitric Oxide (NO)

Nitric oxide (NO), an intercellular messenger, is generated by the enzyme NO synthase and activates soluble guanylyl cyclase leading to cyclic guanosine monophosphate (cGMP) formation. In turn, cGMP-activated protein kinases regulate multiple substrates such as DARPP-32 and G-substrate, which inhibits phosphatases that are involved, among other effects, in synaptic plasticity expression (for review see: Hardingham et al., 2013). Concerning STDP, in the somatosensory cortex of mice, Hebbian tLTP depends both on the α-amino-3-hydroxy-5-methyl-4-isoxazolepropionic acid receptor (AMPAR)-subunit-1 and a NO-dependent presynaptic component (Hardingham and Fox, 2006). Similarly, glutamate afferents to serotoninergic neurons of the dorsal raphe nucleus exhibit tLTP for pre-post pairings, which is NO-dependent, involving the cGMP-activated protein kinase signaling cascade (Haj-Dahmane et al., 2017). In retinal ganglion cells of tadpoles, STDP can be induced by natural visual stimulation (e.g., moving bar) or by electrical stimulation of the retina and in both cases, NO is required for tLTD while BDNF is required for tLTP (Mu and Poo, 2006).

### GABA

**Figure 3:**
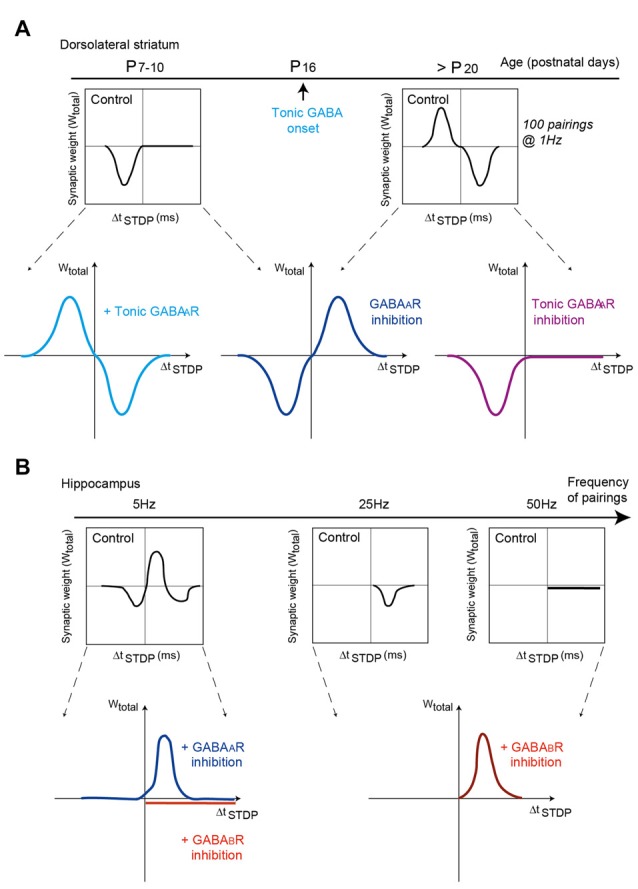
GABA_A_ and GABA_B_ receptor activation shapes STDP in dorsal striatum and hippocampus. **(A)** Modulation of striatal synaptic plasticity by GABAergic signaling at different post-natal ages. Schematic view of the impact of GABAergic signaling on corticostriatal STDP throughout development. Left, at P_7–10_, inhibition of GABAergic signaling turns Hebbian tLTD into bidirectional Hebbian STDP. Selective activation of tonic GABAergic signaling converts Hebbian tLTD into bidirectional anti-Hebbian STDP (as observed at P_25–30_). Adapted from Valtcheva et al. (2017). Right, at P_25–30_, inhibition of GABAergic signaling shifts bidirectional anti-Hebbian STDP into bidirectional Hebbian STDP. Selective inhibition of tonic GABAergic converts bidirectional anti-Hebbian STDP into Hebbian tLTD (as observed at P_7–10_). Adapted from Paille et al. (2013) and Valtcheva et al. (2017). **(B)** In hippocampus, depending on the frequency of STDP pairings (5, 25 and 50 Hz), inhibition of GABA_A_ or GABA_B_ receptors shape differently STDP expression and polarity. GABA_A_ receptors modulate the timing dependence of tLTD whereas GABA_B_ receptors control STDP frequency dependence. Adapted from Nishiyama et al. (2010) and Sugisaki et al. (2016), with no permission required.

In the dorsal striatum, anti-Hebbian STDP as observed in control conditions in striatal output neurons is shifted to Hebbian STDP under pharmacological blockade of GABA_A_R receptors (Fino et al., 2010; Paille et al., 2013; Valtcheva et al., 2017; Figure 3A). This effect applies equally at D_1_R-class striatopallidal (direct pathway) and D_2_R-class striatonigral (indirect pathway) neurons of juvenile and adult rodents. Although the molecular mechanisms underneath this reversal of polarity by GABA are not fully elucidated, a computational model suggests that depolarizing effects of GABA at distal dendrites would reverse calcium influx by modifying the balance between calcium influxes from NMDAR vs. voltage-sensitive calcium channels (Paille et al., 2013). Although GABA increases calcium influxes in both NMDAR and voltage-sensitive calcium channels, via its depolarizing effect in striatal output neurons (due to the relative values of the chloride reversal and membrane potential), the depolarizing effect of GABA would impact differentially NMDAR and voltage-sensitive calcium channels depending on the order of pairings (post-pre vs. pre-post). GABA would favor calcium influx via voltage-sensitive calcium channels for post-pre pairings (promoting tLTP), whereas it would favor calcium influx via NMDARs for pre-post pairings (promoting tLTD) in control conditions, leading to anti-Hebbian STDP (Paille et al., 2013). Under GABA blockade, this balance between calcium influxes is shifted and Hebbian STDP can be observed. Change in GABAergic signaling during striatal development (i.e., the onset of the tonic GABAergic signaling around P_14_; Ade et al., 2008) appears to be a key factor for shaping of striatal STDP. Indeed, in young rats (P_7–10_) corticostriatal STDP is unidirectional and Hebbian (tLTD with post-pre pairings, no plasticity with pre-post pairings) but bidirectional and anti-Hebbian in adult rodents (Valtcheva et al., 2017; Figure 3A). GABA signaling is also at play with the control of CB_1_R-dependent tLTP which expression shifts from post-pre to pre-post stimulation when ionotropic GABA_A_ transmission is blocked (Cui et al., 2015). GABA is not involved in the induction of STDP *per se*, nor its magnitude, but controls STDP polarity, i.e., the association between the sign of the pairing (pre-post or post-pre) and the plasticity outcome (tLTP or tLTD). The tonic GABAergic component plays a major role in the emergence of the anti-Hebbian striatal STDP in juvenile and adult rodents (Valtcheva et al., 2017; Figure 3A). Thus, the pathological deregulation of tonic GABAergic signaling may affect the polarity and occurrence of striatal plasticity and alter procedural learning and memory. It remains to be seen whether the neuromodulator role of GABA for STDP emergence and/or polarity constitutes a general rule in the brain.

Change of STDP polarity induced by GABAergic transmission has also been observed in hippocampus. In hippocampal CA1 pyramidal cells, blockade of GABA_A_Rs converts unidirectional tLTD to bidirectional Hebbian STDP (with 80 pairings at 5 Hz; Sugisaki et al., 2016; Figure 3B). The modulatory effects of GABA_A_ and GABA_B_ receptors can also combine. Indeed, at Schaffer collateral-CA1 excitatory synapses of the rat hippocampus, plasticity relies on postsynaptic GABA_A_ receptors to set the spike-timing dependency and also depends on presynaptic GABA_B_ receptors for its frequency dependence (Nishiyama et al., 2010; Figure 3B). Specifically, postsynaptic GABA_A_ receptors regulate the timing dependence of tLTD at 5 Hz pairings (in the theta frequency band), whereas presynaptic GABA_B_ receptors control the frequency dependence of tLTD at 25 Hz (alpha and beta frequencies) and also accounts for the expression of tLTP for 5 Hz and 50 Hz (gamma frequencies; Nishiyama et al., 2010). In addition, STDP can be expressed at GABAergic interneurons, where it modulates the strength of GABAergic inhibition since STDP pairings alters the activity of potassium-chloride cotransporter-2, resulting in changes in the reversal potential of GABAergic synaptic currents (Woodin et al., 2003).

Taken together, the above results indicate that the spectrum of the third factor of STDP is very large since in addition to neuromodulators it can be extended to BDNF, NO and neurotransmitters acting as neuromodulators such as GABA. STDP synaptic plasticity is thus modulated, whether in its induction, its direction or its temporal window. Though neuromodulation of STDP has been investigated for the early phase of plasticity (within the first hour, i.e., the induction phase), the effects of neuromodulators remain to be investigated for the late phases of plasticity in which the third factor is expected to have a crucial role for the maintenance of memory (Lisman et al., 2011).

### Modulation of STDP by Astrocytes: the Forgotten Third Factor

Many forms of excitatory STDP rely on either pre- or postsynaptic glutamate receptors (Sjöström et al., 2008; Feldman, 2012; Korte and Schmitz, 2016). Therefore, STDP is expected to be tightly controlled by glutamate dynamics. Specifically, the spatiotemporal profile of glutamate may define the extent and location of recruited glutamate receptors, which are involved in the induction of tLTP or tLTD.

An overriding question is how coincident synaptic activity in the millisecond range can be integrated over a longer timescale during the iteration of pre- and postsynaptic pairings to allow STDP induction, while keeping sharp sensitivity to timing during individual pairing episodes. A potential solution to this problem could be that: (1) glutamate should be released in a delayed manner to allow integration of pre- and postsynaptic activity over the time course of minutes; and (2) synaptically released glutamate during neuronal activity needs to be reliably cleared from the extracellular space to allow high fidelity sampling of coincident pre- and postsynaptic activity during STDP pairings. Astrocytes help solve this problem of controlling extracellular glutamate dynamics and have been shown to play an important role in synaptic transmission, as well as short- and long-term memory (Chung et al., 2015; Oliveira et al., 2015). This role of astrocytes has led to the concept of the tripartite synapse, comprised of the pre- and postsynaptic neuronal elements as well as the astrocytes. Indeed, a substantial part of central synapses are contacted by astrocytes (Bernardinelli et al., 2014). Notably, astrocytes are able to release glutamate via exocytosis in response to neuronal activity (Araque et al., 2014; Sahlender et al., 2014; Verkhratsky et al., 2016) and to efficiently clear glutamate from the extracellular space on a submillisecond timescale via high-affinity glutamate transporters (Danbolt, 2001). Therefore, astrocytes can both detect and control neuronal activity via the release and reuptake of glutamate.

Astrocytes can integrate the coincident neuronal activity during STDP pairings and participate in the induction of tLTD (Min and Nevian, 2012). Excitatory tLTD induced by post-pre pairings at layer 4 onto layer 2/3 synapses in the rat barrel cortex relies on the release of endocannabinoids by the postsynaptic element through the activation of astrocytic CB_1_Rs (Min and Nevian, 2012). In turn, glutamate released by astrocytes activates presynaptic NMDARs which are required for tLTD induction (Rodríguez-Moreno and Paulsen, 2008). Astrocytes are able to sense postsynaptic endocannabinoid release by gradually increasing their calcium waves exclusively during repetitive post-pre pairings within a narrow temporal window of Δt_STDP_ = −25 ms which is eligible for tLTD induction. Indeed, pre-post pairings at Δt_STDP_ = +25 ms and post-pre pairings at Δt_STDP_ = −250 ms, which induce tLTP and no plasticity, respectively, do not trigger any changes in calcium dynamics (Min and Nevian, 2012). Therefore, astrocytes are selective to a unique temporal pattern, which both generates calcium dynamics to promote glutamate release and imposes a threshold for tLTD induction. Astrocytes can thus act as a time buffer by integrating coincident pre- and postsynaptic activity over the time course of minutes and enabling tLTD by delayed release of glutamate.

Astrocytes also are crucial for the gating of both tLTP and tLTD in the dorsal striatum via the uptake of glutamate (Valtcheva and Venance, 2016). Physiological activity of the astrocytic glutamate transporter, called the excitatory amino acid transporter-2 (EAAT2), allows the expression of bidirectional anti-Hebbian STDP induced in a narrow temporal window −30 < Δt_STDP_ < +30 ms (Fino et al., 2010; Paille et al., 2013; Valtcheva and Venance, 2016). When EAAT2 is blocked, a form of LTP that does not rely on coincident detection can be induced by uncorrelated activation of pre- and postsynaptic elements. This non-Hebbian LTP requires postsynaptic back-propagating action potentials and extrasynaptic GluN2B-containing NMDARs, which are activated by glutamate spillover. In contrast, the overexpression of EAAT2 prevents the expression of striatal STDP (Valtcheva and Venance, 2016) possibly by restricting glutamate availability for both the NMDARs and mGluRs required for striatal STDP (Shen et al., 2008; Fino et al., 2010). Thus, preserving the optimal temporal contingency between pre- and postsynaptic activity required for STDP depends on astrocytic glutamate uptake. Astrocytes gate tLTP and tLTD by a subtle regulation of the extracellular glutamate levels and, therefore, a precisely tuned range of EAAT2 activity allows the emergence of STDP. Computational models have begun to explore interactions between glutamatergic synapses and astrocytes (De Pittà et al., 2011; De Pittà and Brunel, 2016 see also De Pittà et al. (2012) for a review), but investigating the role of astrocytic glutamate control requires transforming the binary glutamate release event typically used in STDP models into glutamate diffusion and update mechanisms.

Astrocytes can release various other neurotransmitters and factors besides glutamate (Araque et al., 2014; Sahlender et al., 2014; Verkhratsky et al., 2016) including the NMDAR co-agonist D-serine which regulates different forms of synaptic plasticity. The release of D-serine is necessary for frequency-dependent LTD and LTP in the hippocampus (Zhang et al., 2008; Henneberger et al., 2010) and prefrontal cortex (Fossat et al., 2011). Moreover, experience-dependent changes in the degree of synaptic enwrapment by astrocytes governs the level of D-serine availability and subsequently controls the expression of NMDAR-dependent LTP and LTD in the supraoptic nucleus of the hypothalamus of lactating rats (Panatier et al., 2006). The NMDARs implicated in STDP can be situated at both pre- or postsynaptic sites (Feldman, 2012; Korte and Schmitz, 2016) and thus may be affected to different extents by gliotransmission. D-serine has a permissive role for the induction of NMDAR-dependent tLTP at mossy fiber-CA1 hippocampal synapses (Rebola et al., 2011), although its glial origin has not been investigated. In the developing hippocampus a presynaptic tLTD at CA3-CA1 synapses requires D-serine signaling possibly released from astrocytes (Andrade-Talavera et al., 2016). Interestingly, the same STDP pairing protocol induces tLTP at later developmental stages suggesting the possibility that astrocytic coverage of neurons and modulation of STDP by gliotransmission may be developmentally regulated.

Another important gliotransmitter is adenosine triphosphate (ATP), which is enzymatically converted to adenosine in the extracellular space and can act on pre- and postsynaptic adenosine receptors situated on neurons. Glial release of ATP controls the magnitude of hippocampal LTP induced with high-frequency stimulation (Pascual et al., 2005) and blockade of postsynaptic adenosine A2a receptor increases the amplitude of low-frequency stimulation-dependent LTD in the striatum (Lerner et al., 2010). Adenosine also mediates striatal tLTP via postsynaptic adenosine A2a receptors both *in vitro* (Shen et al., 2008) and *in vivo* when the STDP paradigm is coupled with dopamine pairing (Fisher et al., 2017). In addition, presynaptic adenosine A1 receptors modulate the amplitude of tLTP in the visual cortex (Bannon et al., 2017). However, evidence directly implicating astrocytes in the purinergic control of STDP is still lacking. Computational models of signaling pathways underlying STDP have begun to include adenosine A2a receptors (see below), but investigation of interaction between pre-synaptic NMDA and adenosine A1 receptors requires modeling of mechanisms controlling pre-synaptic vesicle release.

Finally, astrocytes are involved in the GABAergic modulation of both the polarity (Fino et al., 2010; Paille et al., 2013; Valtcheva et al., 2017) and threshold for induction (Groen et al., 2014) of excitatory STDP. Astrocytes regulate basal and transient inhibitory tone via GABAergic transporters (Scimemi, 2014). Non-specific blockade of both neuronal and astrocytic GABA transporters in the developing striatum has a permissive role for the induction of tLTD (Valtcheva et al., 2017) but the particular contribution of astrocytic GABA clearance in STDP remains to be explored.

## Molecular Pathway-Based Computational Models of Stdp

**Figure 4:**
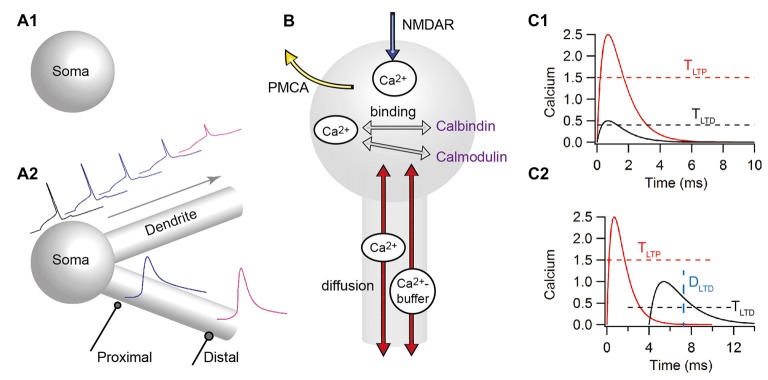
Computational models for predicting the direction of STDP have a wide range of complexity. **(A)** Models differ in morphological complexity, from single-compartment **(A1)** to multi-compartment models **(A2)**. Top traces show that the back-propagating action potential decreases in amplitude, initiates later and broadens as it propagates distally in multi-compartmental models. Bottom traces show that distal synapses may produce higher calcium elevations than proximal synapses due to higher local input resistance. **(B)** Models differ in the mechanisms used to control calcium dynamics, from single time constant of decay, to biophysical/biochemical models of diffusion (red arrows), pumps (such as the plasma membrane ATPase: PMCA) that extrude calcium (yellow arrow), buffers (such as calmodulin, calbindin, or immobile buffers) that bind to free calcium (gray arrows) and calcium release (not shown). All models include influx through the N-methyl-D-aspartate receptors (NMDARs; blue arrows). **(C)** The prediction of plasticity from calcium often uses two amplitude thresholds **(C1)**, but sometimes include duration thresholds **(C2)** or other measures of calcium duration. T_LTP_, tLTP amplitude threshold; T_LTD_, tLTD amplitude threshold; D_LTD_, threshold on the duration of the calcium elevation.

In an attempt to better understand the mechanisms governing learning and memory and determine which mechanisms control input-dependent plasticity, modeling efforts have focused on biophysical and biochemical models that utilize a kinetic description of the molecular pathways implicated in STDP. These models range in molecular complexity from single ion (i.e., calcium) to complicated signaling pathways, and in spatial complexity from single-compartment (Figure 4A1) to multi-compartment (Figure 4A2). An overview of this literature can be found in several review articles (see e.g., Graupner and Brunel, 2010; Griffith et al., 2015). In the following, we focus on the articles published after 2010, though include the most influential contributions published before that date. Moreover, in the following, we subdivide the models into two types: those evaluating the control of plasticity from calcium dynamics alone, and those that add one or more downstream signaling pathway molecules. In addition, we try to distinguish single-compartment models from those that add some degree of spatial structure to the postsynaptic neuron. We acknowledge that in both of these dimensions the classification is not binary and some models bridge the divide.

### Simplified Calcium Dynamics and Two-Threshold Rules

Models of calcium dynamics in response to STDP stimuli are the most common type of models, and are justified both by the critical role of calcium in plasticity and also by the stimulation protocol in which neuromodulator release does not change. The only difference between STDP protocols that produce tLTP and STDP protocols that produce tLTD is the timing between the presynaptic stimulation and the postsynaptic action potential, Δt_STDP_; thus the number and frequency of presynaptic stimulations does not differ between tLTP and tLTD. This implies that presynaptic release of neuromodulators does not differ so it must be postsynaptic molecules activated by calcium dynamics that determine the polarity of plasticity.

Calcium predicting the direction of synaptic plasticity is one of the ideas that are popular among theoreticians and experimentalists. In the simplest form the peak calcium (or indeed the amplitude of the current through the calcium permeable, NMDA subtype of the glutamate receptor) controls the direction of plasticity (for reviews see: Graupner and Brunel, 2010; Evans and Blackwell, 2015; Griffith et al., 2015). This is known as the “two-threshold” rule: if calcium (either peak or integrated) is above the higher, potentiation threshold, tLTP is induced, whereas if calcium is larger than the lower LTD threshold but lower than the LTP threshold, tLTD occurs (Figure 4C1). Pre-post pairings produce a large calcium influx through the NMDA receptor channel with calcium concentration above the LTP threshold, whereas post-pre pairings produce a moderate calcium influx with calcium concentration between the LTD and LTP thresholds. One of the first models of NMDAR-dependent synaptic plasticity was proposed by Shouval et al. (2002). This model, using simplified calcium dynamics inside a dendritic spine, accounted for a diverse range of stimulation protocols such as STDP and classical rate-based plasticity; however it predicted depression for long positive Δt_STDP_, a model prediction which is not confirmed by experiments (but see Nishiyama et al., 2000, 2010; Wittenberg and Wang, 2006). In the dorsal striatum, a model of calcium dynamics (Evans et al., 2012) evaluated the role of NMDAR subunit (2A and 2B subunits) in shaping the sensitivity to timing dependence, and correctly predicted that NMDAR-2A would require a small Δt_STDP_, whereas NMDAR-2B can support tLTP with a large Δt_STDP_. Several extensions or modifications to the basic model have been made both to account for results with spike triplets (i.e., when either two presynaptic stimuli or two postsynaptic action potentials are generated) and to minimize the tLTD window for long positive spike-timings. Adding another coincidence detection of presynaptic NMDARs with endocannabinoids is one mechanism utilized in a neuromorphic implementation of calcium based synaptic plasticity (Rachmuth et al., 2011). Alternatively, incorporating short term depression of transmitter release or AP back-propagation (Shouval and Kalantzis, 2005; Bush and Jin, 2012) minimizes the tLTD seen with long positive Δt_STDP_ and can account for other experimental results; however, a more broadly applicable study (Rubin et al., 2005) showed that plasticity rules that use calcium amplitude alone cannot completely avoid predicting tLTD for long positive timings.

An extension of the two-threshold rule states that the duration of calcium elevation is equally important in determining direction of plasticity (Figure 4C2). Several models of STDP explicitly take into account both the amplitude and the duration of calcium in predicting plasticity outcome (Kumar and Mehta, 2011; Graupner and Brunel, 2012). Including a duration threshold or integrating the total calcium response allows correctly predicting experimental outcomes for both traditional STDP curves and STDP curves produced by spike triplets. Another extension of the Shouval et al. (2002) model, Standage et al. (2014), implements a calcium-dependent, sigmoid-shaped time constant of calcium decay, which represents saturation of calcium extrusion from the spines. This model shows that saturation of calcium extrusion might be responsible for the dependence of tLTP on the (theta-frequency like) inter-spike interval for triplet stimulation protocols. Including the duration of calcium does not exclude consideration of presynaptic release probability on STDP. Indeed, gliotransmission may change the shape of the STDP curve depending on whether gliotransmitters increase or decrease presynaptic release (De Pittà and Brunel, 2016).

### Threshold Rules Based on Detailed Calcium Dynamics

Most of the aforementioned models use simplified calcium dynamics instead of explicitly implementing the mechanisms underlying control of calcium (Figure 4B), which might improve predictions of synaptic plasticity. In other words, the next set of models used neither single time constant of decay nor summation of independent pre- and postsynaptic components for calcium dynamics. Though not explicitly implementing a STDP rule, Griffith et al. (2016) indirectly consider the effect of calcium duration by using calcium-bound calmodulin to assess how back-propagating action potential timing influences calcium concentration. Using a 3-dimensional, deterministic reaction-diffusion model of calcium interactions with calmodulin and other calcium binding proteins within a dendritic spine, Griffith et al. (2016) show that calcium-bound calmodulin is a more sensitive indicator of spike timing than free calcium. They further demonstrate the role of neuromodulators in regulating synaptic plasticity through their activation or inhibition of calcium dependent potassium channels during an STDP protocol, which greatly modulates calmodulin activation.

Several studies explicitly investigate how the dendritic location and inhibitory inputs shape the local calcium-based plasticity rules (Bar-Ilan et al., 2013; Jędrzejewska-Szmek et al., 2016). Bar-Ilan et al. (2013) showed that inhibition shapes the spatial profile of dendritic calcium concentration in neocortical pyramidal neurons. Depending on the location of the excitatory and inhibitory inputs on the dendritic tree (Figure 4A2), tLTP may be blocked, transformed to tLTD, or the synapse may undergo no plasticity. Similarly, Jędrzejewska-Szmek et al. (2016) developed a computational model of the major neuron type in the striatum, the striatal output neurons, including both electrical activity and calcium dynamics. They demonstrated that calcium amplitude and duration together (Figure 4C2) can predict a wide range of experimental plasticity outcomes, and further demonstrated a distance dependence of STDP caused by the back-propagating action potential. In both of these models, the distance dependent decreases in back-propagating action potential amplitude reduces calcium influx through NMDARs for more distant synapses. This reduced calcium influx can convert tLTP into either tLTD or no plasticity. These publications demonstrate that by modeling mechanisms controlling calcium dynamics, including diffusion, buffers and pumps, and by considering calcium duration, the LTD window for long positive Δt_STDP_ is avoided.

An aspect of calcium dynamics often ignored in modeling studies is calcium release from intracellular stores. This has been shown to contribute to tLTP under some conditions (Plotkin et al., 2013; Cui et al., 2016). Thus, Nakano et al. (2013) included calcium release from stores in their multi-compartmental model of a direct pathway spiny projection neuron. In addition, though not explicitly including other signaling pathways, they evaluated the effect of dopaminergic modulation of calcium, potassium and NMDAR channels. The main result of their simulations showed that dopaminergic input preceding a back-propagating action potential induced higher calcium responses than dopamine input following a back-propagating action potential. This study also predicted that the timing dependence of calcium responses between the up- and down-states was similar.

### Models of Signaling Pathway to Explain Synaptic Plasticity

Beyond calcium, several models add on simplified or abstract version of downstream signaling molecules. Rubin et al., 2005 propose a three detector system, loosely based on pathways resembling the opposing Ca^2+^/calmodulin-dependent protein kinase-II (CaMKII)—protein phosphatase signaling pathways. In brief, three calcium-sensitive detectors are implemented: high, transient calcium levels activate the tLTP detector; low calcium elevations activate the tLTD detector; and intermediate calcium levels activate a “Veto” detector. Another variable integrates both the tLTD detector and the Veto detector (called a double filter), such that intermediate calcium levels decrease the double filter value; thus the double filter detects the uninterrupted duration of calcium at low values yet suppresses the development of tLTD should calcium spend some time at intermediate values, such as occurs with long positive Δt_STDP_. Using the three calcium detector system of Rubin et al. (2005), Cutsuridis (2011) showed that single GABAergic inhibitory inputs can sharpen the shape of the STDP curve: narrowing the temporal window that supports tLTD, whereas a train of GABAergic inputs both sharpens the tLTD window and reduces the tLTP amplitude. A follow-up study (Cutsuridis, 2012) extended the model to burst stimulation, and predicted that GABAergic inputs would expose a tLTD window for long positive Δt_STDP_. The timing of the GABA inputs determined whether the effect was predominantly depression or potentiation.

**Figure 5:**
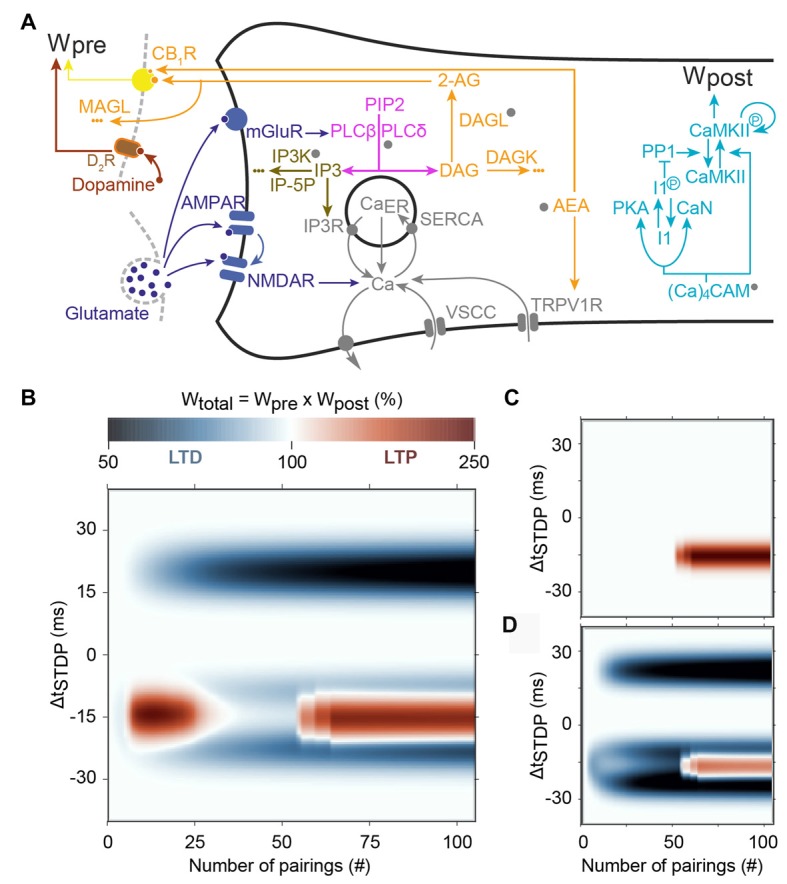
Main predictions of the model of Cui et al. (2016). **(A)** Scheme of the signaling pathways that are considered in the model. The postsynaptic weight is set by the amount of phosphorylated CaMKII whereas the presynaptic weight is controlled by the activation of cannabinoid type-1 receptor (CB1R). Abbreviations: PIP2, phospatidylinositol 4,5-biphosphate; DAG, diacylglycerol; IP3, inositol-1,4,5-triphosphate; PLCβ/δ, phospholipase-Cβ/δ; DAGLα, diacylglycerol lipase α; 2-AG, 2-arachidonoylglycerol; AEA, anandamide; TRPV1, transient receptor potential cation channel subfamily V member 1; IP3R, IP3-receptor channel; SERCA, sarcoplasmic/endoplasmic reticulum calcium ATPase; Ca_ER_: calcium in the endoplasmic reticulum; (Ca)_4_CaM, fully bound calmodulin; CaN, calcineurin aka PP2B; PKA, protein kinase A; I1p/I1, phosphorylated/unphosphorylated protein phosphatase-1 inhibitor 1 (DARPP-32 in striatal output neurons); PP1, protein phosphatase 1; CaMKII, Ca^2+^/calmodulin-dependent protein kinase II. **(B)** Prediction of the evolution of the total synaptic weight (product of the pre- and postsynaptic weights) when the spike timing and the number of pairing varies. tLTD progressively emerges at positive Δt_STDP_, whereas for negative Δt_STDP_, the model correctly predicts two domains of tLTP, one around 10–20 pairings and another emerging after 50 pairings. **(C)** When CB_1_R are blocked in the model, both the tLTD and the tLTP for low pairing numbers disappear. **(D)** Adding presynaptic D_2_Rs in the model, correctly predicts that tLTP for low pairing numbers is also controlled by dopamine. Adapted from Cui et al. (2016) with no permission required.

Several models (Graupner and Brunel, 2007, 2012; Pi and Lisman, 2008; Carlson and Giordano, 2011; Mihalas, 2011; Saudargienė and Graham, 2015; Cui et al., 2016; for reviews see: Graupner and Brunel, 2010; Evans and Blackwell, 2015; Griffith et al., 2015) have implemented even more realistic representations of signaling pathway kinetics, including the calcium activated phosphatase calcineurin, the calcium activated kinase, CaMKII, and the Gs-activated adenylyl cyclase, the latter of which produces cAMP to activate PKA (Figure 5). Additional pathways, such as PKC (resulting from activation of Gq-coupled receptors such as M_1_R and mGluR) and extracellular signal-regulated protein kinase (ERK; downstream of protein kinases A, C and tyrosine receptor kinase B) are also involved. Several advantages accrue from these models, including the ability to produce experimentally testable predictions regarding the role of specific molecules. Another key advantage of simulating signaling molecules is that the tLTD window for long positive Δt_STDP_ is eliminated without arbitrarily assuming the existence of a dedicated calcium concentration range that does not elicit synaptic plasticity, i.e., a separate range between the tLTD-inducing calcium range and the tLTP range. Again these models vary in complexity, such as the number of different signaling pathways included, and whether spatial aspects are included. Several models of these signaling molecules have been applied to STDP protocols in the cortex, hippocampus and striatum.

One of the earliest models, the single-compartment electric model of Graupner and Brunel (2007), couples membrane potential with a biochemical reaction model via calcium dynamics. Phosphorylation state of CaMKII serves as the models readout, i.e., the level of phosphorylated CaMKII serves as a proxy of the synaptic weight. Short positive intervals can switch the CaMKII to a highly phosphorylated state; whereas negative intervals (but not long positive intervals) switch the CaMKII to a low phosphorylated state. Critical to success of this model is adjustment of calcium dependence of PKA and calcineurin activity against inhibitor 1, which controls the level of free protein phosphatase 1. A high-level of protein phosphatase 1 will dephosphorylate CaMKII to prevent its persistent activation. Indeed, in this model (Figure 5): (i) the protein phosphatase-1 (PP1)/CaMKII activation ratio dictates plasticity; LTD is expressed when PP1 activation overcomes CaMKII, whereas LTP occurs when CaMKII activation is larger than PP1 activation; and (ii) PP1 activity is maximal at intermediate calcium levels whereas CaMKII activation needs larger calcium levels. Short negative Δt_STDP_ yield intermediate but long lasting calcium levels, which efficiently activate PP1 but are not large enough to activate CaMKII, thus triggering LTD. Short positive Δt_STDP_ yield sharp calcium peaks that are large enough to activate CaMKII but do not persist long enough around intermediate values to activate PP1 significantly; this leads to LTP. Finally, the calcium levels triggered by long positive Δt_STDP_ are too weak to activate CaMKII but do not stay long enough around intermediate values to activate PP1. Long positive Δt_STDP_ therefore fail to activate either the PP1 or CaMKII, which in effect rules out the expression of tLTD. This molecular system therefore exhibits dynamics similar to the “Veto” detector proposed by Rubin et al. (2005) to eliminate tLTD at long positive spike timings (see above).

Subsequent models either enhance the electrical activity model, or add AMPA receptors as a model readout. Urakubo et al. (2008) develop a multi-compartment, multi-ion channel model of visual cortex pyramidal neurons to activate a biochemical reaction model. In contrast to Graupner and Brunel (2007), the timing dependence of tLTD cannot be reproduced unless calcium-bound calmodulin allosterically inhibits NMDARs. Both Carlson and Giordano (2011) and Saudargienė and Graham (2015) used a model of AMPAR insertion controlled by the CaMKII/protein phosphatase-2A switch. Carlson and Giordano (2011) used a single-compartment model of calcium dynamics (from Shouval et al., 2002) to activate the biochemical network model of Pi and Lisman (2008). This single-compartment model can explain STDP and does not predict tLTD for long positive Δt_STDP_. Voltage-sensitive calcium channels are critical for the latter effect, as blocking voltage-sensitive calcium channels allow tLTD to emerge for long positive Δt_STDP_. Saudargienė and Graham (2015) incorporated spatial aspects of calcium dynamics by using a detailed compartmental model of pyramidal CA1 neuron (Poirazi et al., 2003) to activate a biochemical network model derived from two earlier models (Graupner and Brunel, 2007; Pi and Lisman, 2008). Saudargienė and Graham (2015) showed, by monitoring AMPAR phosphorylation by the CaMKII/protein phosphatase-2A switch, that tLTD is indeed induced by lower calcium levels than tLTP, and that tLTD also requires many more repetitions of this lower calcium (which is consistent with experimental results). Saudargienė and Graham (2015) also investigated the influence of particular timings of inhibition associated with excitatory inputs, showing that inhibition affects tLTD more that tLTP, because tLTD occurs for moderate calcium levels and is thus more vulnerable to any reduction in peak calcium.

Whereas spatial models of calcium dynamics typically include dendritic branching or explicit spines (microdomains), many signaling molecules are anchored via structural proteins into multi-protein complexes, effectively creating nanodomains of molecule interactions. One method for evaluating the effect of nanodomains (without explicitly creating a spatial model) is to couple different sources of calcium to different downstream signaling molecules. This approach was utilized by Mihalas (2011) who coupled three different calcium sources to three different signaling molecules: NMDAR to CaMKII, voltage-sensitive calcium channels to calcineurin, and phosphodiesterase to calcium release. Adenylyl cyclase was coupled to both voltage-sensitive calcium channels and NMDAR. The change in synaptic weight was calculated from kinase (tLTP) and phosphatase (tLTD) activity. This model investigated the role of cAMP degradation in triplet-based STDP, and showed that, if cAMP activity is spatially restricted to the membrane, the STDP profile is similar to that observed in cortical layer 2/3 slices. The STDP profile for spatially diffuse cAMP activity was consistent with that observed in hippocampal cell culture.

In the striatum, endocannabinoid production and activation of CB_1_Rs are required for most forms of tLTD (Mathur and Lovinger, 2012); thus, Cui et al. (2016) extended the signaling pathways from Graupner and Brunel (2007) with 2-arachidonoylglycerol; (2-AG the main endocannabinoid) production via mGluR- and M_1_R activation. Cui et al. (2016) utilized a single-compartment model of electrical activity of a spiny projection neuron for calcium dynamics, coupled with a model of signaling pathways underlying STDP in striatum, including calcium-induced calcium release from internal stores. This model used a combined 2-AG- and CaMKII-based plasticity rule, where the direction of plasticity (LTP or LTD) was determined by the product of the presynaptic weight (2-AG-based) and postsynaptic weight (CaMKII based). The strength of this model is the ability to show the mechanism whereby decreasing the number of pairings converts NMDAR-dependent tLTP to an endocannabinoid-dependent tLTP, which was confirmed experimentally (Figure 5; Cui et al., 2015, 2016). The underlying hypothesis of this model (that was confirmed experimentally) is that moderate activation of CB_1_R caused endocannabinoid-mediated tLTD whereas large CB_1_R activation leads to tLTP. In the model, 10–20 negative pairings trigger large endocannabinoid transients that result in endocannabinoid-mediated tLTP. However, CB_1_R desensitization and partial depletion of calcium in the endoplasmic reticulum (Ca_ER_) starts to be significant after 20 pairings, so that CB_1_R activation is in fact smaller with more than 20 pairings than with 10–20 pairings. As a result, the expression of endocannabinoid-mediated tLTP is restricted to 10–20 negative pairings, in agreement with experimental observations (Cui et al., 2015, 2016). On the other hand, as in the original model by Graupner and Brunel (2007), calcium levels become large enough to activate significant amounts of CaMKII only after 40–50 negative pairings, thus restricting the expression of NMDAR-dependent tLTP to this range of pairings. As a result, this model successfully reproduces the experimental observation that the endocannabinoid-mediated tLTP expressed at 10–20 positive pairings disappears, to be replaced by NMDAR-dependent tLTP after 50 pairings (Figure 5). The addition of presynaptic dopamine signaling to the model correctly predicted that the CB_1_R-dependent tLTP observed with 10–20 pairings is also under the control of presynaptic D_2_R (Figure 5).

### Exploring *in Vivo*-Like Conditions

One benefit of computational modeling is the ability to isolate a specific aspect of STDP and address the impact of this very aspect at the level of networks and/or learning *in vivo*. For instance, Kempter et al. (1998) used spike-based models to explore how the pulse structure of neuronal signals and events on a millisecond scale influenced learning rules. Clopath et al. (2010) utilized a voltage-based plasticity rule, consistent with a wide body of experimental data, to study the emergence from plasticity of connectivity patterns in a cortical network. Along the same line, variants of the classical computational STDP rule have been devised that yield broad synaptic weight distributions matching the available experimental observations (Gilson and Fukai, 2011).

However, one major detractor of STDP is its deterministic and constant spike timing (interval between spikes within a pairing) and inter-stimulation interval (interval between consecutive pairings), which diverge highly from biological variability. One of the most pressing questions of learning and memory is which stimuli resemble *in vivo*-like conditions best. Gjorgjieva et al. (2011) showed that a triplet model of STDP, depending on the interactions of three precisely timed spikes, described plasticity experiments closer to natural stimuli measured in the brain. Graupner et al. (2016) compared *in silico* plasticity outcomes to several types of irregular, *in vivo*-like, firing patterns to investigate the influence of firing rate and spike timing on synaptic plasticity. They showed that sensitivity of plasticity to spike-timing is reduced by adding jitter (irregularity) to spike-pairs. Using physiological firing patterns recorded in awake behaving macaque monkeys, Graupner et al. (2016) further showed that moderate variation of firing rate, without any timing constraints, could reproduce synaptic changes induced by spike timing. This result offers a different view on the central role played by spike timing in long-term synaptic plasticity.

Most computational models of STDP indicate that plasticity disappears when the timing between pre and postsynaptic pairings loses its regularity. However, it is not clear what amount of noise can be tolerated for STDP or ITDP to be expressed (robustness) and whether this amount depends on the signaling pathway supporting the plasticity. This question has recently been tackled both experimentally and in a computational model, using noisy STDP stimulations where the timing between the pre- and the postsynaptic stimulations was jittered (Cui et al., 2018). As stated above, in striatum three forms of STDP are observed: NMDAR-tLTP, endocannabinoid-tLTD and endocannabinoid-tLTP (Cui et al., 2015, 2016). These three forms do not show similar sensitivity to jittered spike timing: NMDAR-tLTP appeared poorly resistant whereas endocannabinoid-plasticity (tLTD and tLTP) appeared more robust (Cui et al., 2018). Moreover, increasing the average pairing frequency or the number of pairings reinforces NMDAR-tLTP and increases resistance to jittered spike timing. These results suggest that the probability to observe the various forms of STDP *in vivo* is a multivariate function of the mean spike timing, the number of pairings, the frequency of pairings and also the variability of the spike timing. The shape of this multivariate function is thus more complex than e.g., a monotonic decay with increasing variability of the spike timing, and could reveal a functional specialization of each of these STDP forms to sub-regions of the stimulation train parameters.

## Conclusions and Future Directions

In addition to the pre- and postsynaptic firing patterns, a third factor for STDP control comprises not only the classical neuromodulators (dopamine, noradrenaline or acetylcholine to name a few) but also neuropeptides (BDNF), unconventional neurotransmitters (NO) and astrocytes surrounding neurons, which can uptake or release neurotransmitters and neuromodulators. The spectrum of the third factor of STDP is even larger since it can be extended to neurotransmitters acting as neuromodulators such as GABA and glutamate (via their tonic component) and endocannabinoids. Here, we reviewed the main effects of the third factor on STDP: from the emergence of STDP, to the shaping of STDP i.e., the dependence on Δt_STDP_, and the magnitude and polarity of plasticity.

Beyond the time scale of Δt_STDP_ that is consistently in the ~80 ms range, the studies that explored STDP properties have used a large variety of pairing protocols to induce STDP. This diversity in stimulation protocol renders the comparison between studies exceedingly difficult. As described above, beside its dependence on Δt_STDP_, STDP expression is highly affected by varying the structure of STDP pairings (1:1, 1:2, … n:n or theta bursts; Edelmann et al., 2015), or the number and/or frequency of pairings (Sjöström et al., 2001; Cui et al., 2016) (for review see Sjöström et al., 2008; Feldman, 2012; Edelmann et al., 2017). It is thus expected that the effect of neuromodulation also would strongly depend on the STDP activity pattern (as an example see Edelmann et al., 2015).

How the local interneuron networks (GABAergic or cholinergic) or the neuromodulatory afferents are recruited and impact STDP, depends on the activity patterns of the two main inputs. i.e., the third factor effect may vary with or depend on a triplet of characteristics: Δt_STDP_, number of pairings, frequency of pairings. Optogenetics will most certainly be a key method to induce neuromodulator release in a more time-controlled manner to mimic for example phasic activity or explore precisely the retroactive action of neuromodulation on STDP properties.

The number of experimental studies investigating the signaling pathways underlying the STDP expression and their modulation by a third factor is still limited and needs further consideration. The signaling pathways underlying frequency-dependent plasticity (triggered by high- or low-frequency stimulations) have been more thoroughly explored, but need to be fully address in STDP. Signaling though G-protein coupled receptors is far more complex than the static view of the list of proteins that compose each signaling pathways. For instance, G-protein coupled receptors exhibit the “biased agonism”, i.e., the notion that a given agonist of a signaling pathway activates only a subset of all the signaling pathways associated with its receptor (Kenakin and Christopoulos, 2013). In other words, two agonists of the same signaling pathway, even of the same receptor, activate different subsets of reactions, thus yielding different biological effects. One potential mechanism explaining biased agonism is the interplay between differential ligand-binding kinetics and the kinetics associated with different cell signaling processes (Klein Herenbrink et al., 2016). In this context, the subsets of signaling processes effectively activated by STDP pairings could differ from those activated by the stronger protocols employed in frequency-dependent plasticity.

The complexity of G-protein coupled receptor signaling also has consequences on STDP modulation. The available experimental data surveyed above point to a general rule according to which neuromodulation by monoamines or acetylcholine is mostly controlled by the type of G-protein coupled receptors activated: regardless of the agonist, G_i_-coupled and G_q/11_-coupled receptors favor tLTD, whereas Gs- and Golf-coupled receptor activation leads to tLTP. One might therefore erroneously conclude that two modulators would have the same effect by activating the same signaling pathway. This would of course be at odds with the concept that different neuromodulators exhibit different biological effects, due to different receptor affinities, different receptor locations, co-localization of diverse downstream signaling molecules, and the ability of phosphorylated receptors to switch their coupling to different G proteins. Hence, dopamine signaling via D_1_R may display different biological effects from noradrenaline signaling via β–adrenergic receptors, although both activate the G_s_/G_olf_ signaling pathway. Future computational models of STDP modulation should aim to reconcile the general scheme of the above rule with the specificity of neuromodulators, probably through variants of biased agonism.

Because of the complexity of the mode of action of neuromodulators, most of the studies have investigated the role of only a small number of neuromodulators one by one (the neuromodulator systems have mostly been activated or inhibited one-at-a-time), but the crosstalk between neuromodulators is critical, as demonstrated in only a few studies: for dopamine and acetylcholine (Brzosko et al., 2017), dopamine and GABA (Xu and Yao, 2010), dopamine and noradrenaline (Seol et al., 2007) or dopamine and endocannabinoids (Cui et al., 2015). The effects of other neurotransmitters/neuromodulators (such as adenosine, serotonin or endocannabinoids), or neuropeptides (substance P, enkephalins, oxytocin), fatty acids (arachidonic acid, cholesterol, omega-3), hormones or the role of other non-neuronal cells (astrocytes, oligodendrocytes, microglia, pericytes, ependymal cells or endothelial cells) remain to be investigated in STDP expression; Indeed, most of these actors are known to modulate rate-dependent plasticity. Furthermore, the effects of neuromodulators in STDP maintenance remain to be determined and not only for the induction phase of STDP. It has been shown in a rate-coded plasticity at CA1 hippocampal synapses that D_1_-like-receptor inhibition blocks late-phase LTP (Huang and Kandel, 1995), impedes consolidation of memory and accelerates its erasure (Wang et al., 2010; Lisman et al., 2011). Similarly, the third factor effect should be evaluated in the late phase of STDP (maintenance and potentially erasure).

By fully taking into account the third factor, i.e., a multicomponent learning rule, the computational power of neural networks might be considerably improved (as reviewed in Kuśmierz et al., 2017). Up to now, the third factor has usually been considered in isolation from the pre- and postsynaptic firing patterns. This experimental convenience might well disguise more complex network-level properties. In this regard, the fact that the level of tonic GABA in the local network can switch STDP from Hebbian to anti-Hebbian may have important consequences in dendritic computation and in a network context (Hiratani and Fukai, 2017). The interplay between changes of the firing rate of some of the network neurons due to Hebbian STDP and resulting changes in tonic GABA could give rise to abrupt STDP shifts locally from Hebbian to anti-Hebbian. Such local STDP shifts may provide the network with self-organizing properties that would not be predicted easily when the third factor is considered in isolation. Added to the fact that different synapse types in the network can have different STDP rules (and possibly, different modulation by the third factor), the complexity and variety of the resulting network dynamics would considerably increase. Note that here again, computational models will be instrumental to explore the potential impact of these mechanisms on the dynamics and functional properties of neural networks.

A fair criticism of the physiological relevance of STDP has been raised by Lisman et al. (2011) since *in vivo* the back-propagating action potential is obviously not triggered with a somatic current injection in the postsynaptic neuron (as classically performed in STDP experiments) but rather with the dynamic integration of synaptic inputs whose build-up would eventually reach the action potential threshold. Input-timing-dependent plasticity (ITDP), a form of heterosynaptic plasticity, consists in paired activation of presynaptic inputs separated by an interval Δt_ITDP_, leading to sub- or suprathreshold activity in the postsynaptic neuron (Dudman et al., 2007; Williams et al., 2007). Therefore, ITDP could be viewed as an attractive naturalistic upgrade of STDP, not only for experimental studies (Dudman et al., 2007; Cho et al., 2011; Mehaffey and Doupe, 2015; Brandalise et al., 2016; Leroy et al., 2017) but also for computational models (Shim et al., 2016). ITDP has been reported in amygdala following activation of thalamic and cortical inputs (Humeau et al., 2003; Cho et al., 2011), in hippocampal CA1 (Dudman et al., 2007), CA2 (Leroy et al., 2017) or CA3 (Brandalise et al., 2016) pyramidal cells and in avian basal ganglia (Mehaffey and Doupe, 2015). Interestingly, GABA and enkephalin have been shown to modulate CA2 hippocampal ITDP (Leroy et al., 2017), which paves the way for future studies investigating the role of the third factor in ITDP properties.

The vast majority of STDP studies investigating the third factor have been achieved *ex vivo* (cell cultures or acute brain slices), although few studies have addressed neuromodulation of STDP *in vivo* (Mu and Poo, 2006; Schulz et al., 2010; Cassenaer and Laurent, 2012; Yagishita et al., 2014; Fisher et al., 2017). In *ex vivo* studies, neuromodulators (dopamine, acetylcholine) are typically applied exogenously because of their very low levels when compared to *in vivo*. Neuromodulators are released in tonic and phasic modes *in vivo* and therefore *ex vivo* bath-applications of neuromodulators or specific agonists hardly mimic such complexity of the neuromodulation. It would be important to explore the *in vivo* neuromodulation needed to stabilize STDP or ITDP, by transforming eligibility traces into plasticity, and thus allowing an activity pattern sequence to be pertinent for the engram. Thus, there is a need to collect data *in vivo* in awake and behaving animals and model *in vivo*-like plasticity rules and stimulation patterns to fully understand the action of the third factor in Hebbian learning and information storage and recall.

## Author Contributions

AF and AM participated in the writing of the “Neuromodulators Affecting the Expression Polarity and Shape of STDP” section. JJ-S, HB and KB wrote the “Molecular Pathway-Based Computational Models of STDP” section. SV wrote the “Modulation of STDP by Astrocytes: the Forgotten Third Factor” section. LV wrote the “Introduction, Neuromodulators Affecting the Expression Polarity and Shape of STDP and Conclusion” sections. All authors have edited and corrected the manuscript.

## Conflict of Interest Statement

The authors declare that the research was conducted in the absence of any commercial or financial relationships that could be construed as a potential conflict of interest.

## Abbreviations

AMPAR: α-amino-3-hydroxy-5-methyl-4-isoxazolepropionic acid receptor
ATP: adenosine triphosphate
BDNF: brain-derived neurotrophic factor
cAMP: cyclic adenosine monophosphate
CaMKII: Ca^2+^/calmodulin-dependent protein kinase-II
CB_1_R: cannabinoid type-1 receptor
cGMP: cyclic guanosine monophosphate
DARP-32: dopamine- and cAMP-regulated phosphoprotein, Mr 32 kDa
D_X_R: dopaminergic type-X receptor
EAAT2: excitatory amino acid transporter-2
ERK: extracellular signal–regulated kinase
GABA: gamma-aminobutyric acid
ITDP: input-timing dependent plasticity
mAChRs: muscarinic acetylcholine receptors
mGluR: metabotropic glutamatergic receptor
M_X_: muscarinic type-X receptor
nAChRs: nicotinic acetylcholine receptors
NMDAR: N-methyl-D-aspartate receptor
NO: nitric oxide
STDP: spike-timing dependent plasticity
tLTD: timing-dependent long term depression
tLTP: timing-dependent long term potentiation.
